# Implementing and evaluating an e-portfolio for postgraduate family medicine training in the Western Cape, South Africa

**DOI:** 10.1186/s12909-019-1692-x

**Published:** 2019-07-08

**Authors:** Magdaleen De Swardt, Louis S. Jenkins, Klaus B. Von Pressentin, Robert Mash

**Affiliations:** 1 Division of Family Medicine and Primary Care, Department of Family and Emergency Medicine, Faculty of Medicine and Health Sciences, Stellenbosch University, PO Box 241, Cape Town, 8000 South Africa; 2 Mossel Bay District Hospital, Mossel Bay, South Africa; 3 George Regional Hospital, George, South Africa; 4 Garden Route District, Western Cape Department of Health, Cape Town, South Africa

**Keywords:** E-portfolio, Family medicine, Postgraduate, Training, Assessment, Workplace

## Abstract

**Background:**

In South Africa it is compulsory to submit a satisfactory portfolio of learning to gain entrance to the national exit examination of the College of Family Physicians and to qualify as a family physician. A paper-based portfolio has been implemented thus far and the need for an electronic portfolio (e-portfolio) was identified. The aim of the study was to describe and evaluate the implementation of an e-portfolio for the training of family medicine registrars in the Western Cape province of South Africa.

**Methods:**

Mixed methods were used. A quasi-experimental study evaluated paper- and e-portfolios from the same 28 registrars in 2015 compared to 2016. Semi-structured interviews were conducted with 11 registrars or supervisors to explore their experiences of using the e-portfolio. Quantitative data was analysed in the Statistical Package for Social Sciences and qualitative data in Atlas.ti.

**Results:**

Most respondents found the e-portfolio easier to use and more accessible. It made progress easier to monitor and provided sufficient evidence of learning. Feedback was made easier and more explicit. There were concerns regarding face-to-face feedback being negatively affected. It was suggested to have a feedback template to further improve feedback. Several aspects were significantly better in the e-portfolio such as feedback on the registrar’s general behaviour, alignment with learning outcomes, less feedback based on hearsay and acknowledgement of the feedback by the registrar. Although not statistically significant, there was an increase in the usage of the e-portfolio, compared to the paper portfolio.

**Conclusion:**

In general, the e-portfolio is an improvement on the paper-based portfolio. It is easier to access, more user-friendly and less cumbersome. It makes feedback and monitoring of progress and development of registrars easier and more visible and provides sufficient evidence of learning. Its implementation throughout South Africa is recommended.

**Electronic supplementary material:**

The online version of this article (10.1186/s12909-019-1692-x) contains supplementary material, which is available to authorized users.

## Background

Worldwide, high stakes assessment of postgraduate medical training now incorporates workplace-based assessments, with regular observations, evaluation and feedback, to improve the validity of performance assessment [1–4]. Miller’s pyramid for categorising approaches to assessment puts workplace-based assessment at the pinnacle as it evaluates what registrars actually do in the real world setting [5]. Complex competencies can be observed more easily in the workplace, although with less standardisation, than in a simulated environment such as an objective structured clinical examination (OSCE).

A portfolio of learning is increasingly recognized as reliable and valid evidence of an individual’s personal and professional development. If implemented correctly, with registrars and supervisors understanding its purpose, it provides an authentic assessment of performance in the workplace [6–8]. The College of Family Physicians of South Africa made the submission of a satisfactory portfolio of learning compulsory for registrars to qualify for the national exit examination [9]. The portfolio, together with the national exit examination (three written papers and a 20-station OSCE) and a research assignment, are needed in order to qualify as a specialist family physician. Subsequently, a national paper-based portfolio of learning for the discipline was successfully developed, implemented and validated [10–13].

Internationally there is a shift from using paper-based portfolios to using web-based electronic portfolios (e-portfolios) [14–16]. While the use of e-portfolios is now embedded in many high-income countries, it is still an emerging educational tool in low and medium income countries, such as South Africa [17]. The advantages of e-portfolios above paper-based portfolios include being more user-friendly, less cumbersome, more manageable with flexible access and content that is easier to keep up to date [18]. E-portfolios cannot be lost and supervisors can assess progress of learners from any internet connection on a regular basis. E-portfolios are more efficient at giving feedback and encourage reflection in a legible format [4, 7, 19–21]. The disadvantages of e-portfolios compared to paper-based portfolios are the need for reliable internet access, stable, high quality and expensive technological infrastructure and users who are sufficiently skilled in using the software and hardware [22].

In South Africa the public health system takes care of the health needs of 84% of the population, while 16% of the population have private health insurance [23]. The district health system, consisting of 52 districts, has been identified by the National Health Act (Act 61 of 2003) as the vehicle through which 90% of all public health care should be delivered [24]. The country is suffering from huge inequities and a quadruple burden of disease, which includes maternal and child care, human immune deficiency virus (HIV) and tuberculosis, interpersonal violence, and non-communicable diseases [25]. Consequently, the National Department of Health has embarked on a process of establishing a National Health Insurance (NHI) scheme and re-engineering of the primary health care (PHC) system, in order to provide universal health coverage, improve equity, strengthen capacity and the quality of health care. This scheme includes family physicians as a key component [26].

Since the Health Professions Council of South Africa recognized family medicine as a specialty in 2007, the various roles of the family physician have been clarified [27]. These include being a clinician, consultant, capacity builder, clinical trainer, taking a lead in clinical governance and supporting community orientated primary care [27–29]. The National Human Resources for Health Plan emphasizes the role of the family physician as the clinical leader of the multi-professional team in each health district [30]. Recent work indicates that just over 200 family physicians work in the public sector in South Africa [31]. With the shortage of health care professionals in the country, the National Human Resource for Health policy aims to increase the number of family physicians in the public sector to 1000 by 2020 [28]. This has training capacity implications, as many more family medicine registrars need to be trained [32, 33].

It is essential that family medicine registrars are appropriately trained, supervised and assessed, to ensure they acquire the necessary competencies for their roles and responsibilities in the health system. Summative and formative assessments occur in relation to the national learning outcomes and a clinical skills list [32, 33]. Registrars are trained in accredited training complexes, over 4 years, with rotations through regional and district hospitals and primary care facilities. Clinical training is workplace-based and teaching also occurs through direct interaction on campus as well as through online, web-based modules. Evidence of learning is provided through workplace-based assessments, captured in portfolios, and formal examinations.

The shift of focus to workplace-based assessments by means of a portfolio of learning coupled with increasingly better access to the internet and use of web-based educational platforms for distance education (e.g. Moodle, Blackboard) enabled the development of an e-portfolio [12, 13]. Some programmes are utilising the on-line course management software, while others are looking at software designed specifically for portfolios of learning. The Colleges of Medicine of South Africa are looking at software that can be used across all colleges.

There has been some work done in Africa on e-portfolios, for example among teachers [34] and institutions for higher learning [35]. In South Africa, a paper-based portfolio of learning for postgraduate family medicine training was developed and implemented nationally during 2010 [10–13]. This involved workshop training in its use at all nine medical schools, being accepted for the national exit examination by the College of Family Medicine of South Africa, and regular updating of the online version. Apart from this work and searching the literature on the use of portfolios for postgraduate training for Family Medicine in Africa, the authors could not find other published work in this field on the continent. This identified the specific knowledge gap this paper attempts to address. The aim of this research was to evaluate the introduction and use of an e-portfolio for postgraduate family medicine training at Stellenbosch University in South Africa. The objectives included:

1. To describe the introduction and implementation of an e-portfolio of learning for registrars in the Western Cape.
2. To compare the utilization of the e-portfolio with the paper-based portfolio.
3. To compare the quality of interaction and feedback from supervisors in the e-portfolio with the paper-based portfolio.
4. To describe the process and challenges in migrating from the paper-based portfolio to an e-portfolio.
5. To recommend how the e-portfolio can be improved upon and its use expanded to other health faculties in South Africa and Africa.

## Methods

### Study design

A convergent mixed methods design was used, which included a quasi-experimental study as well as semi-structured interviews with registrars and supervisors.

### Setting

This study was conducted among family medicine registrars and their family physician supervisors in the Western Cape during 2015 and 2016. The Division of Family Medicine and Primary Care in the Faculty of Medicine and Health Sciences at Stellenbosch University trains registrars towards a MMed (FamMed) degree in five training complexes, of which four are rurally situated. These include Eden (now renamed Garden Route), West Coast, Cape Winelands, Overberg, and Metro-East. At the time of the study there were 36 registrars in training and 38 electronically registered potential supervisors. Supervisors with access to the e-portfolio included the family physicians as well as other specialists from the regional hospital that might provide clinical training.

An e-portfolio of learning was designed and piloted during January to April 2016 in the Eden training complex and introduced in May 2016 to all the complexes in the Western Cape. A collaboration was established with Mateum©^,^ a software company based in Maastricht, in the Netherlands. Their software package, called E-PASS©, was adapted with the support of a local educational company, Intaka©, based in Cape Town, to form the e-portfolio [36]. The local e-portfolio design was based on the previous paper-based portfolio and had the same content and structure (see Table 1). Registrars and supervisors outside of the Eden complex only received training on use of the e-portfolio during April 2016 and had to retrospectively upload learning activities that took place between January and April 2016.

**Table 1:** Contents and structure of the portfolio

Section	Requirement	Paper-based	Electronic-based
1	Introduction: this section explained the use of the portfolio.	Printed series of pages.	File attachment accessed via a “documents” hyperlink.
2	Learning outcomes: this section presented the national learning outcomes, what was assessed in the portfolio and what the requirements were.	Printed series of pages that also highlighted what was expected in the portfolio for each outcome.	File attachment accessed via a “documents” hyperlink. The landing page (dashboard) dynamically compared what had been submitted with what was expected.
3	Learning plans and reflections on learning: each registrar was required to submit a minimum of two learning plans and two reflections on their learning per year.	Completed template and assessment from the supervisor which could be hand written or typed and inserted in file.	Learning plans revised through a series of electronic iterations. Final version was approved by supervisor. Reflections on learning submitted via template and assessed by supervisor.
4.	Educational meetings: A minimum of 24 h / year and a variety of learning conversations: A: Leadership and clinical governance B: Clinical care C: Family and community orientated care D: Teaching and training others E: Professionalism and Ethics F: Other	Date, type of learning conversation, duration in hours and description filled in on a printed form and signed off by supervisor.	Filled in online on a template and approved by supervisor.
5	Observations by supervisor of consultations, procedures or teaching: A minimum of 10/year.	Multiple copies of the observation tools included in the portfolio for use by the registrar and supervisor.	Templates online and filled in and approved by supervisor. PDF copies available and can be printed out or sent via e-mail and filled out by hand if supervisor does not have access to portfolio.
6	Written assignments: A minimum of two assignments per year. Assignments were completed and marked in SunLearn, but then added to the portfolio.	Assignments printed out and added to file.	Assignments uploaded and validated by supervisor.
7	Logbook: Consists of a list of core clinical skills that needs to be acquired during the four year training programme and needs assessment at least twice a year. A: Only theory B: Seen or have had demonstrated C: Apply/Perform under supervision D: Independent	Printed series of pages, each clinical skill needs to be assigned an A, B, C or D and discussed / assessed by supervisor and signed with comments and date.	Template online with a logbook for each domain and each domain needs to be discussed and validated by supervisor separately. Option to register (enter) a tally of individual procedures performed.
8	A certificate of training in resuscitation and life support is a requirement to sit for final examination.	Certificates can be added to the portfolio.	Certificates can be scanned and uploaded as a file.
9	Other courses, congresses, workshops, lectures.	Proof can be inserted in this section of file.	Proof can be scanned and uploaded as a file.
10	End of year assessment: Portfolio Assessment Tool (PAT) (see below: Table 2).	Assessor must look through the whole portfolio and extract the data needed to complete the assessment.	Assessor views the automatically collated scores in the template and adds a final score and any feedback.

The primary researcher (MdS) graduated from the University of the Free State and was a final year registrar in Family Medicine at the University of Stellenbosch in 2017. She used the paper portfolio since 2014 and took part in the pilot study of the e-portfolio in the beginning of 2016 and used the e-portfolio during 2016 and 2017.

#### The quasi-experimental study

##### Sample size and sampling

All registrars in their second, third and fourth year of training and their supervisors were invited to participate. Participants’ use of the paper-based portfolio in 2015 was compared to their use of the e-portfolio in 2016. First year registrars in 2016 and fourth year registrars in 2015 were excluded because they did not have any comparative data. The study population was 28 registrars and since all registrar participated, no sampling was necessary. As this was not a case-control study, no matching was necessary. There were no participants lost to follow-up.

##### Data collection

Data was collected from the paper-based portfolios and e-portfolios by using the eight indicators which was derived from a previously validated portfolio assessment tool (see Table 2): [9].

**Table 2:** Portfolio assessment tool (PAT)

	Indicators in the portfolio	Score or grading in the portfolio	Description of indicators	Minimum needed in the portfolio/year
1	Learning plans	/10	Mean rating of the written learning plan by the supervisor.	6-monthly
2	Reports on performance	/10	Mean rating of the registrar’s performance by the supervisor.	6-monthly
3	Educational meetings	/20	Number of hours accumulated scored out of 10 and the range of different types of educational interactions scored out of 10.	24 h, 5 different types of interactions
4	Observations by supervisors	/10	Mean rating of the registrar performing different competencies such as a consultation, procedure or teaching event.	10 observations
5	Assignments	/10	Mean of grades obtained for written assignments.	2 assignments
6	Logbook	/30	Rating of competency to perform each clinical skill by the supervisor adjusted to score out of 30.	2 ratings per year
7	Global rating	/10	Rating of the overall evidence of learning, quality of reflection and organisation of the portfolio.	Once a year
8	Total grade	/100		

The portfolio required at least two learning plans and two reports on performance, each with an average weighting of 10%. Furthermore, a minimum of 24 h of educational meetings per year, with a weighting of 20% were expected. Ten observations by supervisors (assessed via mini-Clinical Evaluation Exercise (mini-CEX) or Direct Observation of Procedural Skills (DOPS)) carried 10% weighting, and assignments from online modules carried another 10%. The logbook needed to be completed for a number of procedures [30] and carried a 30% weighting. The final global assessment score by the programme manager made up the last 10% of the portfolio assessment. If these requirements were met the portfolio was deemed complete. To qualify for the final exams at the end of four years training, three completed portfolios were needed.

By using the indicators in Table 2, the paper- and e-portfolios of registrars were compared in terms of the completeness of the portfolio (minimum entries needed in portfolio) and quality of their entries (score or grading) as well as the overall assessment of the portfolio (total grade). The portfolios were also compared in terms of the number of entries per month as a measure of the frequency of use and ongoing engagement. This would give an indication whether the portfolios were only completed at the end of the year, when required for summative assessment purposes, or used as an ongoing stimulus and record of learning. A tool was developed to record and evaluate the frequency and quality of feedback given by the supervisors (see Additional file 1). The content of the tool was informed by the literature and content validity derived from an expert panel consisting of four experts in medical education, family medicine and clinical training. The quality criteria used in the tool included: general impression of performance, feedback on clinical behaviour, positive and negative valence of feedback, strategies for improvement, and the means by which feedback was obtained (direct observation or hearsay). The scoring and analysis were kept simple with no entry scoring zero and the presence of an entry scoring one.

##### Data analysis

Data was analysed with the Statistical Package for Social Sciences (SPSS®) version 25 [37]. Descriptive statistics were reported as either frequencies and percentages or medians and interquartile ranges as the data was not normally distributed. Inferential statistics were used to compare scores between paired data using the Wilcoxon signed rank test.

#### Semi-structured interviews

##### Sample size, sampling and data collection

Ten registrars and five supervisors were purposefully selected. We aimed to recruit two registrars and one supervisor from each complex and from different years of study, who would be willing to give rich descriptions of the use of their portfolios and allow for data saturation to be achieved. The exploratory question was, “How did you experience the e-portfolio this year?” The primary researcher (MdS) conducted the interviews using an interview guide (see Additional file 1) to explore participants’ views on the technical aspects of the e-portfolio, the overall utility, the impact on learning and teaching and any difficulties they experienced. The interviews were conducted in privacy at a time convenient for the study participants, and audio-taped, with field notes. All interviews were in English and there was no need for translation.

##### Data analysis

The interviews were transcribed verbatim, checked for accuracy and analysed according to the Framework method [38] and with the help of Atlas-ti® version 6.2.27 [39] using the following steps: familiarisation with the data by going through the transcripts multiple times; identifying a thematic index of codes, coding the transcripts; charting/grouping the data from the same codes; and interpreting the data to identify themes, with appropriate quotations. Themes were deductively identified by the primary researcher (MdS) and verified by the first supervisor (LSJ). These results were also triangulated with the quantitative data from the quasi-experimental study to strengthen the overall evaluation.

## Results

### Quantitative results

The portfolios of 28 participants were included, with an equal split between males and females (see Table 3), although there were more rural than metro-based participants.

**Table 3:** Demographics of portfolio participants

Year of study in 2016			Rural vs. Metro		Total
			Rural	Metro	
First Year	Gender	Male	5	0	5
		Female	2	2	4
	Total		7	2	9
Second Year	Gender	Male	0	2	2
		Female	3	2	5
	Total		3	4	7
Third Year	Gender	Male	3	0	3
		Female	1	1	2
	Total		4	1	5
Fourth Year	Gender	Male	1	3	4
		Female	3	0	3
	Total		4	3	7
Total	Gender	Male	9	5	14 (50%)
		Female	9	5	14 (50%)
	Total		18 (64%)	10 (36%)	28

The demographics of the study participants, namely the equal split between male and female registrars, can be explained by the profile of people selecting the study programme and subsequently being trained. The reason for the 18/10 rural/metro split is because there are 4 rural training complexes and one metro training complex in the overall programme. While the training programme context across the platform have many sub-district variations, the basic institutional structures and educational contents of the various sites are similar, all being at district and primary health care level, allowing for reasonable comparisons.

In terms of completeness of the portfolios, there were only four incomplete e-portfolios per year group, either due to dropping out or only starting half way through the year, and this was not statistically significant when compared with the paper-based portfolios.

The evaluation of the different portfolio sections was not significantly different between the paper-based and e-portfolio (see Table 4).

**Table 4:** Comparison of portfolio assessment scores

Section of the portfolio assessment	2015 paper Median (IQR)	2016 electronic Median (IQR)	*p*-value
Learning plan (/10)	8.65 (6.17–10.0)	7.00 (4.25–8.0)	0.152
Supervisor report (/10)	8.00 (7.37–8.63)	8.00 (6.5–8.7)	0.624
Educational meetings (/20)	19.2 (16.0–20.0)	18.0 (15.95–20.0)	0.790
Number of observations	10.0 (10.0–10.0)	10.0 (7.25–10.0)	0.180
Observations score (/10)	7.7 (7.0–8.4)	8.0 (7.8–8.44)	0.317
Assignments (/10)	7.3 (6.85–7.8)	7.0 (6.0–8.0)	0.398
Logbook (/30)	25.3 (20.0–28.0)	29.0 (24.75–30.0)	0.625
Global rating (/10)	6.0 (6.0–7.0)	6.0 (5.0–7.0)	0.418
Score for reflections	3.0 (2.0–3.0)	2.0 (2.0–3.0)	0.142
Total score (/100)	77.3 (62.75–86.23)	83.0 (76.0–86.75)	0.221

**Fig. 1:**
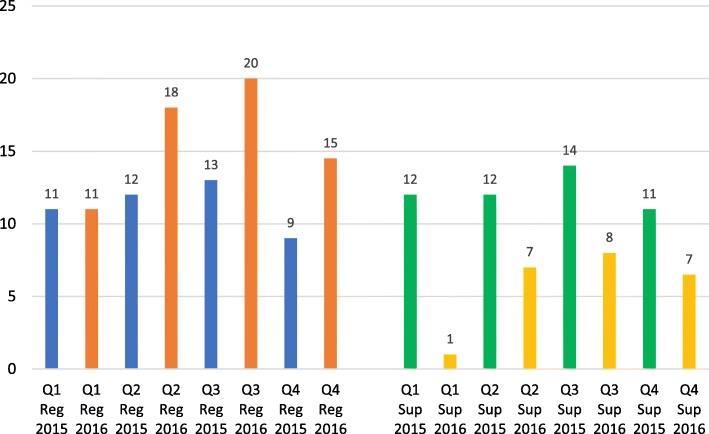
Median number of portfolio entries per quarter for registrars (reg) and supervisors (sup)

While not statistically significant overall, the registrars’ number of quarterly portfolio entries increased from the paper- to the e-portfolio. The supervisors, on the other hand, showed a decrease in their quarterly portfolio entries (see Fig. 1). The median comparison using the non-parametric test showed statistically significances for quarters 1, 2 and 3 for the supervisors only (*p*-values of 0.005, 0.035 and 0.016, respectively). The comparisons for the registrars were not statistically significant.

Table 5 compares the quality of feedback between the paper-based- and e-portfolios. Several aspects were significantly better in the e-portfolio such as feedback on the registrar’s general behaviour, alignment with learning outcomes, less feedback based on hearsay and acknowledgement of the feedback by the registrar. Feedback on specific behaviour and highlighting of areas for improvement were improved in the e-portfolio and might have been statistically significant if the study had more power. The total number of words provided in the feedback increased from a median of 350 words in the paper-portfolio to a median of 583 words in the e-portfolio, which was not statistically significant, but suggested a trend towards improvement in feedback.

**Table 5:** Comparing the quality of feedback

Feedback assessment	2015 paper Median (IQR)	2016 electronic Median (IQR)	*p*-value
1. Feedback in general (non-specific)	14 (14.0–14.0)	14 (14.0–14.0)	0.344
2. Feedback on registrar’s behaviour			
2.1 General behaviour	11 (8.0–12.0)	13 (12.0–13.0)	0.040*
2.2 Specific behaviour	6 (3.0–8.0)	8 (6.0–11.0)	0.079
3. Type of feedback			
3.1 Highlights competency	12 (8.0–13.0)	12 (10.75–13.0)	0.721
3.2 Highlights deficiencies	1 (0.0–2.0)	4.5 (2.0–6.0)	0.070
4. Feedback on how to improve			
4.1 General suggestions	3 (1.0–5.0)	4.5 (3.75–8.0)	0.481
4.2 Specific suggestions	3 (2.0–5.0)	5 (3.0–7.0)	0.906
4.3 Aligned to learning outcomes	0 (0)	2 (1.0–2.0)	0.001*
5. How was feedback assessed?			
5.1 Based on direct observation	14 (13.0–14.0)	14 (11.0–14.0)	0.137
5.2 Based on hearsay from others	5(3.0–5.0)	2 (1.0–3.0)	0.028*
6. Total number of words	350 (223–599)	583 (462–1042)	0.345
7. Feedback acknowledged	6 (4.0–8.0)	14 (11.0–14.0)	0.009*

*Statistically significant at *p* < 0.05

### Qualitative interviews

Six registrars and five supervisors were interviewed. The demographics of the interviewees were as follows: eight male respondents and three female from all five training complexes. Two registrars from each year group (2nd, 3rd, 4th years) and five supervisors participated. One registrar from Eden, Cape Winelands, West Coast, and Overberg, and two from East Metro participated, while two supervisors from Eden, one from Cape Winelands, West Coast and East Metro, and nil from Overberg participated. The respondents’ ages ranged from 28 to 56 years. Data saturation was thought to have been reached after 11 interviews.

The themes that emerged from the interviews are summarised in Table 6 and elaborated more in Table 7.

**Table 6:** Themes from the interviews

	Themes
1	E-portfolio was simple and accessible
2	E-portfolio helped to improve monitoring of progress
3	E-portfolio made feedback more visible
4	E-portfolio captured evidence of learning iteratively

**Table 7:** Themes with benefits, challenges and suggestions for improvement

Themes	Benefits	Challenges	Suggestions for improvement
1. E-portfolio was simple and accessible.	1. User-friendly 2. Easily accessible 3. Well organised 4. Easy to navigate 5. Continuously available	1. Four learning plan iterations, difficult to conceptualise and not explained clearly enough. 2. Logbook entries could only be entered once and as a block entry per medical discipline, which made revisiting and updating them impossible. 3. Registering of procedures was time consuming with too much detail to fill in. 4. Initial conversion of paper-based data to electronic format mid-way through the year.	1. Explain iterations better, and make clearer in e-portfolio. 2. Allow for easier access to logbook entries 3. Mobile application
2. E-portfolio helped to improve monitoring of progress.	1. Registrars and supervisors interacted more frequently throughout the year and not just at the end of the year. 2. Supervisors and programme managers could monitor progress throughout the year as access to registrars’ portfolio was available all the time, not just at end of year. 3. Registrars could send supervisors a reminder via electronic format to review new additions to their portfolio.	Educational mind shift needed to work on portfolio more regularly.	Ongoing awareness among supervisors to engage with registrars’ portfolios.
3. E-portfolio made feedback more visible.	1. Electronic tracking of feedback became possible, together with the clarity of the entries. 2. Difficult feedback could be given more easily. 3. Registrars had to electronically acknowledge that they read their feedback.	1. Face-to-face feedback was neglected or compromised. 2. Feedback was not specific enough.	More structured template for giving feedback and not just a single open text box.
4. E-portfolio captured evidence of learning iteratively.	1. Easier to add their learning experiences regularly to their e-portfolios, which supported a more iterative developmental process.	1. Still a sense that the purpose of the portfolio was to provide evidence of learning to the faculty rather than enabling the registrar’s own self-development. 2. Constrained by the structure of the e-portfolio.	1. Allow for other media and more memory space and to be able to personalise their e-portfolio more. 2. Allow for more learning plans, reflections and assignments.

#### E-portfolio was simple and accessible

Most registrars and supervisors reported that the e-portfolio was an improvement on the paper-based portfolio:

*“I think it's definitely better. Probably because it is just easier to access wherever you are. You don't really have the ability to forget it unless you forget your mobile device at home.”* (Registrar Interview 2)

All the participants found the e-portfolio user-friendly, easily accessible, well organised, easy to navigate and continuously available. All the participants used their laptops or desktop computers at home to access the e-portfolio and six participants also used their mobile devices, tablets or desktop computers at work:

*“I really liked the whole e-nature of it, the fact that they can do something and it is immediately accessible to us, they don’t have to hand in something physical and then you have to hand it back and so forth and so forth. And any time you know what is waiting for you to assess.”* (Supervisor Interview 11)

*“I liked that it’s simple and accessible, that it is transparent (supervisors and you can see progress) and that it can be used in every setting.”* (Registrar Interview 10)

The challenges that were experienced included the four learning plan iterations, which were intended to enable interaction between the registrar and supervisor as the plan developed, but were difficult to conceptualise and not explained clearly enough. The logbook entries could only be entered once and as a block entry per medical discipline, which made revisiting and updating them impossible. The registering of procedures was time consuming and had too much detail to fill in. The initial conversion of paper-based data to electronic format mid-way through the year added a frustrating additional administrative load to the registrars and supervisors.

*“Overall I would say the experience was good. It was nice to have everything together in one place, as opposed to paper. It was quite a long learning process though. It wasn't completely user-friendly, but then neither was the original portfolio. I think we could have done with some more training sessions, just to navigate us to exactly what is expected. We started off with the paper portfolio and then had to be transferred across to the e-portfolio, which was quite time-consuming.”* (Registrar Interview 5)

Some participants felt constrained by the structure of the e-portfolio and were asking for other media and more memory space and to be able to personalise their e-portfolio more:

“*I think it is difficult to capture reflections on the procedures observed or conversation observed. I'm feeling maybe an option to look at other ways, like audio visual or photographic ways of capturing evidence of learning in a workplace and I think also making sure that what we capture actually is linked up to a learning plan.”* (Supervisor Interview 1)

The e-portfolio facilitated the interaction between supervisor and registrar, largely because of its continuous availability and ease of access. Most participants found the paper portfolio difficult to carry around, bulky and not readily available:

*“I think the communication with supervisors was made easier … because with the paper portfolio if we want to do a supervised consultation or a procedure we have to look for a blank form. Which is not always around and I may have to run around and it often caused a problem. With this one you have a chance with the supervisor. We can do the procedure and whatever and we just have to remember to put it in the e-portfolio and it would be done. That's a good thing, it simplifies the whole process. It's easier to use.”* (Registrar Interview 3)

#### E-portfolio helped to improve monitoring of progress

Supervisors and registrars reported that the monitoring of the registrar’s progress was made easier:

“ … *when I had to look at something it was also quite nice to go from your own profile into the profiles of your registrars to supervise and just to check where they are in terms of the required aspects of the checklist.”* (Supervisor Interview 1)

Registrars and supervisors reported that they interacted more frequently throughout the year and not just at the end of the year. Figure 1 shows that while the registrars did interact more during the 2nd, 3rd and 4th quarter of the year in the e-portfolio, as compared with the paper-portfolio, the supervisors interacted less so, but still maintained a supervisory presence throughout the year. The dashboard, which is the landing page of the e-portfolio, and layout of the e-portfolio served as a reminder to the registrar and supervisor to make more frequent entries as opposed to the paper portfolio:

*“It is very visual, it summarises very nicely what you were doing, and because it's a requirement it forces you to frequently go there and see where we are. It also helped me to identify the areas in which I should do more. It has a summary which helps direct us. It was much more helpful than the paper portfolio, because in my first year I struggled with the paper: every now and then I had to go through everything I have done. It is much superior.”* (Registrar Interview 3)

*“I think that the registrars who would loaf through the year and then suddenly at the end of the year they come to you with this big logbook/portfolio/file: those guys have suddenly realised that now they have to do the work throughout the year. So there is a greatly improved rhythm from that aspect. We don't get this big chunk of work suddenly because of the portfolio deadline. So from my side that is a big relief. It has made the registrars work throughout the year on the ball, portfolio’ specifically. And I think they realised that it is a reflection of their work. From my part I just log on and validate what needs to be validated.”* (Supervisor Interview 7)

#### E-portfolio made feedback more visible

A major theme was the feedback from the supervisor in the learning and development of the registrar. With the paper-based portfolio, it was difficult to always read the feedback, and it was difficult to track whether the registrar had seen the feedback. This was made more explicit with the e-portfolio, where electronic tracking of feedback became possible, together with the clarity of the entries. Also, difficult feedback could be given more easily in the e-portfolio, as opposed to a face-to-face conversation:

*“This depends on your supervisor and also the quality of your relationship. But the e-portfolio makes it easy to have sufficient feedback from the supervisor.”* (Registrar Interview 10)

*“It was also nice, depending on what type of feedback you give. Sometimes it’s difficult to give verbal face-to-face feedback especially if it’s negative.”* (Supervisor Interview 1)

*“I feel my feedback was more direct with regards to written report/feedback.”* (Supervisor Interview 9)

While the e-portfolio was capturing more feedback, many participants felt that face-to-face feedback was neglected or compromised and that feedback was not specific enough. There were positives and negatives to giving electronic feedback:

*“In a positive way I think that, because a lot of the tools prompt you to give feedback, you got to give feedback, I think it is good in that they might get feedback they never got before. The downside is, or maybe it is the e-portfolio itself, because there is this e-communication taking place, they tend to duck under an actual one on one, face to face meeting, there isn’t this pressure to say I must meet, I must make a chance to check in with my supervisor to actually have a one on one conversation, because I still feel that sometimes there is certain feedback that is better done face to face, one on one … So the downside of giving feedback on the e-portfolio is that that said is enough, but the upside is that I think they get more feedback than what they did get before and that it is been recorded and that they can go back to it and see and refer to it.”* (Supervisor Interview 11)

The supervisor-registrar relationship could be potentially compromised if most feedback were happening electronically, without specifically arranging one-on-one educational meetings:

*“It definitely helps you keeping track of what is happening and what is not happening, a bit easier but I’m worried it is taking the place of that one on one and where the relationship is built up more a bit, there definitely needs to be more than just this … ”* (Supervisor Interview 11)

Participants reported that the e-portfolio per se did not necessarily reflect the relationship between the supervisor and registrar adequately:

*“I don’t think the quality of our real-life professional relationship can ever be captured in a portfolio (electronic or otherwise). If I have to look at the state of my current portfolio there is a measurable discordance between our constant real-time feedback and what is reflected to be graded.”* (Registrar Interview 8)

While there was more happening educationally in real life than the portfolio reflected, the portfolio did help to make the training requirements explicit, providing structure for the educational meetings:

*“We didn’t miss out on anything and all the requirements were met. The one-to-one conversation with registrar was quite nice to have it, right there and then so you can edit on it or put in a form or anything and give feedback. It was also nice, depending on what type of feedback you give... It helped to create a structure wherein there were some requirements and some key aspects that needs to be in the portfolio. So it helped us to also make time available or structure time to meet those requirements. It created more opportunities for interaction with registrar.”* (Supervisor Interview 1)

Following on from this, around the challenges of giving effective feedback, supervisors were asking for a more structured template for giving feedback and not just a single open text box:

“*I would like to have it more structured in a positive all known feedback system. Instead of just having one block. I find that most people are busy so their natural inclination is just to say: “You did well.” And that is not feedback. So I would like to either see the things you could be doing differently or better. A framework instead of just a little block, so that people that aren't familiar with feedback processes have a guide to give feedback that is actually feedback. And not either praise or criticism*.” (Supervisor Interview 6)

#### E-portfolio captured evidence of learning iteratively

Registrars found it easier to add their learning experiences regularly to their e-portfolios, which supported a more iterative developmental process. The paper-based portfolios were often completed retrospectively towards the end of the academic year and did not support continuous reflection on learning and assessment. Work that was done and recorded in the e-portfolio could not get lost and evidence of learning such as reflections and feedback were more organised, clear and permanent. However, there was still a sense that the purpose of the portfolio was to provide evidence of learning to the faculty rather than enabling the registrar’s own self-development:

*“To us working with the students on the floor, it actually doesn't matter. We don't need the e-portfolio to know whether you can do something or not. It's a measurement tool providing evidence of supposed learning to universities or people who aren't working with the registrar.”* (Supervisor Interview 6)

A number of suggestions for improvements were made, including:

### Educational

- The learning plan iterations should be better explained.
- The number of learning plans should be increased.
- The reflections on learning should indicate which learning plan was being reflected upon.
- A structured approach to feedback should be added.

### Technical

- The registering of medical procedures should be simplified and only reflect on the ones actually done.
- Include an e-mail prompt to the registrar, once the supervisor has made an entry.
- More memory space to upload audio files, articles, videos, and photos to improve providing evidence of learning.
- Provide a calendar and reminder function.
- Develop a fully mobile application.
- Give access to more supervisors other than family physicians.

## Discussion

Overall the e-portfolio was an improvement on the paper-based portfolio because it was more accessible, user-friendly, secure, structured, enabled better monitoring of progress and improved the quality of feedback. Some of these advantages corroborate previous work [18]. The transition from a paper portfolio to an e-portfolio was not too disruptive and the well-described ‘implementation dip’ was less problematic than expected [14]. We expected some resistance and scepticism, but with workshops clearly explaining the expectations and how to engage the electronic platform, most concerns were adequately addressed, with acceptable uptake of the new tool.

The assessment of the registrar’s performance was similar between the two types of portfolios. This is not surprising, as the portfolio is simply an educational tool, and learning outcomes are more dependent on the local context, the learner and the supervisor, than the tools being used [6]. It is well known that any assessment method, even if less standardized, may have utility, depending on its use [40]. Furthermore, it has been strongly argued that assessment is an educational design problem that needs a programmatic approach [40]. While a portfolio of learning has become standard assessment practice in many programmes worldwide, especially in workplace-based assessment and revalidation, it remains a challenge to provide sufficient evidence of performance or competence [15, 41, 42]. In order to move from assessing performance to assessing professionalism, the next level in Miller’s revised pyramid [43], there is a need to include more methods that rely on qualitative information [40], which imply professional judgement and are most likely incorporated in improved feedback.

While it seemed that the supervisors gave less monthly feedback, this can be partially explained in that until April 2016 the paper portfolio was still being used, and then had to be converted to the electronic format in May 2016. However, they made less feedback entries in the other quantiles also, although there was no significant difference from the paper-based portfolios.

The e-portfolio allowed programme managers to have a global view of feedback, which was not so easy with the paper-based portfolio. It also allowed for comparison between peers and training complexes. Feedback was also seen as a proxy for the supervisors’ involvement in clinical training at the site, giving programme managers the ability to periodically monitor the contribution of supervisors to the e-portfolio throughout the year and not just when the paper version was submitted. The e-portfolio would therefore be useful to give feedback to supervisors on their performance and to compare supervisors with their peers within and between training complexes, assisting with quality assurance of the programme.

Supervisors needed to understand this new way of giving feedback without seeing the reaction or body language of the registrar [14]. One of the 12 tips given for e-tutoring emphasises tutor awareness of this new role, and encourages a forum for tutors to discuss and exchange ideas with each other [16]. This also allows tutors to share their insecurities, and helps to keep the ‘new innovation’ resonating [16]. While face-to-face meetings remain important, it has been recommended to build in regular protected tutor time to read e-portfolio entries and give feedback during working hours [44, 45]. The finding that some participants found the e-portfolio easier than face-to-face meetings to reflect in or give or receive feedback, is well recognised, particularly around difficult learning experiences [46].

Monitoring of registrars’ global progress and development throughout the year was greatly improved by the e-portfolio. With the previous paper-based portfolios, poor performance was only discovered at the end of the year. This is important educationally, and helps to integrate the portfolio with the curriculum and learning in the local context [6].

What has subsequently been added to the e-portfolio is a mapping of the various portfolio entries onto the five unit standards for training of family physicians. This is making the development of the registrar more visible through spider graphs and other graphs. This ability to analyse, synthesise and graphically display information is a strength of the e-portfolio, which has been shown elsewhere [47].

This study adds to the global discourse on the use of e-portfolios for postgraduate training and assessment of healthcare workers and helps to fill the knowledge gap in South Africa and the African continent in this field. It is hoped that this work will stimulate further work around workplace-based assessment in similar resource-constrained learning environments.

### Study limitations

While we aimed to interview ten registrars, six were interviewed, for the following reasons: one registrar did not have a paper-portfolio for 2015, two registrars did not respond, and the other willing registrar was from Eden district, which would skew the representation. However, from the interviews conducted, the same themes kept emerging, indicating a degree of saturation. The registrars and supervisors were not randomly selected, which limits the generalizability of the results. However, it allowed for in-depth information to emerge.

The subjectivity of the primary researcher was handled through regular discussions between the researcher and two supervisors, as well as an awareness of the literature. Being a registrar at the time of doing the interviews minimized potential power imbalances between the researcher and study participants.

The results could be slightly skewed because the paper portfolio was used prospectively for the full year of 2015 and the e-portfolio was only in full use since May 2016, although the registrars were required to include the whole year in their portfolio retrospectively. Validity of the data was increased with triangulating the results between the qualitative and quantitative components of the study.

### Recommendations

While internet connectivity remains a challenge in many, particularly rural, areas, this challenge is gradually being overcome. Walter Sisulu University, which is situated in an extremely rural part of South Africa, has also successfully adopted the e-portfolio. With minor adjustments in the software, it could be useful to training complexes in similar contexts in South Africa and the region. For example, similar training programmes for family medicine have been implemented in Botswana, Malawi, Zambia and Lesotho.

It should be noted that while many data repository systems are available, some free of charge, the e-portfolio is an interactive system, showing progress, and allowing for narrative registrar reflection and supervisor feedback. Since the development and adaption costs from the original EPASS® system to the South African context have now been done, the cost of implementing and maintaining the operational side of the e-portfolio is feasible.

While it takes a commitment in time and funding, this study suggests that the advantages outweigh the disadvantages, even allowing for an implementation dip, where it has been shown that it takes time to implement an e-portfolio and create buy-in from all role players [16]. It is well known that “*introducing portfolios is just like buying shoes: the best choice depends on purpose and a really good fit happens over time, with lots of use and the right give and take by the user*” [48]. Specific recommendations with regards to the e-portfolio include:

- More data space for audio-visual uploads, links and photos to improve providing evidence of learning.
- More space for learning plans, reflections and assignments.
- A feedback template like: What went well? What did not go well? How can you improve?
- Less cumbersome learning plan iterations.
- A fully mobile application.
- Personalizing the portfolio more, for example give an introduction to the portfolio, which registrars can “decorate” and tell more about themselves and their goals/dreams for the year/studies/family medicine or why they chose family medicine.

## Conclusion

The aim of this research was to evaluate the introduction and use of an e-portfolio for postgraduate family medicine training at Stellenbosch University in South Africa. We showed that the e-portfolio is an improvement on the paper-based portfolio. It is easier to access, more user-friendly and less cumbersome. It makes feedback from supervisors, monitoring of progress and development of registrars easier and more visible, and it provides sufficient evidence of learning. With minor adjustments in the software, it could become even better and be useful to training complexes in similar contexts in South Africa and the region.

## Additional file

Additional file 1: Tool for assessing quality of feedback. Interview guide: Comparing e-portfolio with paper portfolio. (DOCX 20 kb)

## Data Availability

Data is available upon request.

## Abbreviations

DOPS: Direct Observation of Procedural Skills
HIV: Human immune deficiency virus
Mini-CEX: Mini-Clinical Evaluation Exercise
MMed (FamMed): Masters Degree in Family Medicine
NHI: National Health Insurance
OSCE: Objective structured clinical examination
PAT: Portfolio Assessment Tool
PHC: Primary Health Care
