# Supplementary material for: Implementing and evaluating an e-portfolio for postgraduate family medicine training in the Western Cape, South Africa
Source: BMC Med Educ. 2019 Jul 8;19:251. doi: 10.1186/s12909-019-1692-x (PMC6615201; doi:10.1186/s12909-019-1692-x)
Supplement: Supplementary file 1 — Tool for assessing quality of feedback. Interview guide: Comparing e-portfolio with paper portfolio. (DOCX 20 kb) [file 12909_2019_1692_MOESM1_ESM.docx]

## **Addendum 1**

Tool for assessing quality of feedback

# **Addendum 2**

Interview guide: Comparing e-portfolio with paper portfolio

1. How did you experience the e-portfolio this year? (What did you like and what did you not like?)

2. How did you find the use of the e-portfolio (navigation, quality of entries, frequency of use, completion of different sections – learning plans, allocations, allocation reports, educational meetings, observations, logbooks)?

3. How did you find the feedback from your supervisor/registrar in the e-portfolio? (quality, quantity, regular)

4. How did the e-portfolio effect your relationship / interaction with your supervisor/registrar?

5. How did the e-portfolio help/not help you with providing evidence of your learning in the workplace?

6. How was the process of changing from the paper- to the e-portfolio (challenges)?

7. What technological difficulties, if any, did you have with the e-portfolio (computer, phone, internet, passwords, saving, printing, uploading, downloading info, learning plan iterations etc.)?

8. How did you access the e-portfolio most of the time (device type, at work, at home)?

9. How would you compare the e-portfolio to the paper-based portfolio (better/worse)?

10. Any suggestions on how to improve the e-portfolio (change features, new features)?
